# 5′ copyback defective viral genomes are major component in clinical and non-clinical influenza samples

**DOI:** 10.1016/j.virusres.2023.199274

**Published:** 2023-11-21

**Authors:** Xing Li, Zhiping Ye, Ewan P. Plant

**Affiliations:** a Laboratory of Pediatric and Respiratory Viral Disease, Office of Vaccine Research and Review, CBER, FDA, Silver Spring, MD, USA

**Keywords:** DVG, Influenza, Disease, Immune response, ANP32

## Abstract

Clinical samples from people with influenza disease have been analyzed to assess the presence and abundance of Defective Viral Genomes (DVGs), but these have not been assessed using the same bioinformatic pipeline. The type of DVG most described for influenza infections (deletion DVGs) differs from the most commonly described DVGs from non-segmented negative stranded viruses (5′ copyback). This could be attributed to either differences between viruses or the tools used to detect and characterize DVGs. Here we analyze several NGS datasets from people infected with different types of influenza virus using the same bioinformatic pipeline. We observe that 5′ copyback DVGs are prevalent in all human clinical samples but not in the cultured samples. To address this discrepancy between clinical and laboratory cultures, we infected cell culture and ferrets with an H5N8 influenza A virus (FLUAV) and analyzed the DVG composition. The results demonstrate that the DVG population is skewed toward 5′ copyback DVGs in the *in vivo* infections and deletion DVGs in the *in vitro* infections. This demonstrates that there are differences *in vivo* genome production and *in vitro* genome production, and this has implications for how the role of DVGs in clinical disease is studied. We also investigate the role the host cofactor ANP32B has in DVG production.

## Highlights

• Influenza disease is associated with the same type of defective viral genome (DVG) observed with other negative-strand RNA viruses.
• The majority of DVGs from clinical influenza samples are copy-back DVGs in contrast to the deletion DVGs widely described from cell culture experiments.
• The production of DVGs in ANP32B-/- mice is significantly reduced and these mice are less susceptible to influenza disease.
• The same virus strain gives rise to different DVG populations *in vivo* and *in vitro*.
• These results indicate that *in vivo* experiments are more relevant for assessing the impact of DVGs in infections than *in vitro* experiments.

## Graphical abstract

**Figure:**
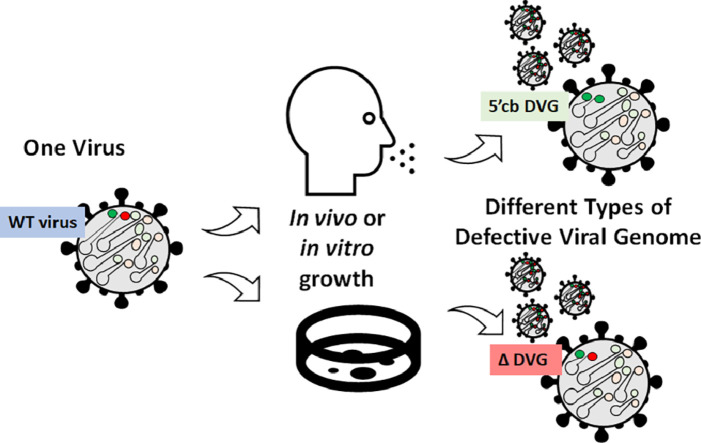

Defective viral genomes (DVGs) have been described in influenza virus preparations from both egg and cell culture but there is less information regarding present DVGs in clinical samples (Rudiger et al., 2021; Von Magnus, 1954). A small number of studies have reported a correlation between disease severity and DVG abundance for influenza and other negative strand viruses (Felt et al., 2021; Lui et al., 2019; Manzoni and Lopez, 2018; Saira et al., 2013; Vasilijevic et al., 2017). We subjected several FLUAV NGS datasets to the same analyses using DVG-profiler (Bosma et al., 2019). These included NGS derived from H1N1, H3N2, H5N1, H5N8 and H7N9 FLUAV subtypes and influenza B virus (FLUBV) (Table 1; (Alnaji et al., 2019; Barnard et al., 2021; Beck et al., 2020; Lumby et al., 2020; Park et al., 2015; Plant et al., 2020; Sobel Leonard et al., 2016; Vasilijevic et al., 2017; Xiao et al., 2019)).

DVG-profiler identifies sequences representing four types of DVG: deletion, insertion, 3′ copyback and 5′ copyback (Bosma et al., 2019). Deletion DVGs are produced when the RNA-dependent RNA polymerase (RdRP) disengages from the template (break point) and re-engages the same template further downstream. Copy-back DVGs occur when the template RNA is replaced with one matching the nascent strand, and the resulting genome has homologous ends, giving the appearance that the RdRP broke from the template and copied back along the strand being generated.

NGS sequences were aligned with the appropriate reference virus using the Hexagon alignment application in the HIVE environment (Santana-Quintero et al., 2014; Simonyan et al., 2016). The alignment length was set at 17 and all matches within acceptable limits were kept. Alignments were analyzed by DVG-profiler using the follow settings: maximum distance of aligned parts, 50; maximum overlap of aligned parts, 5; minimum depth of coverage, 3; minimum length of aligned read, 70 for paired reads and 50 for single reads; minimum length of aligned subject, 20; and the combination with the best score from multiple alignment combinations was kept. DVGS were sorted by type and normalized (unique junction counts per 1000,000 aligned reads). Normalized counts from each sample are graphed and statistical analyses performed using GraphPad Prism version 9.4.1.

We first compared data from FLUAV H1N1 samples cultured in MDCK cells or isolated from patients (Vasilijevic et al., 2017). Both deletion DVGs and 5′ cb DVGs were detected in cell culture samples but were present at much lower levels patient isolated samples (Fig. 1A and B). We next investigated whether DVGs were present in samples from a FLUBV infected patient. We compared the abundance of DVGs from an immunocompromised patient with a persistent FLUBV infection to the DVG populations from laboratory cultured FLUBV samples (Lumby et al., 2020; Plant et al., 2020). In contrast to the FLUAV H1N1 samples, both deletion and 5′ cb DVGs were more prevalent in the clincal FLUBV samples compared to the cultured samples (Fig. 1C and D).

**Fig. 1:**
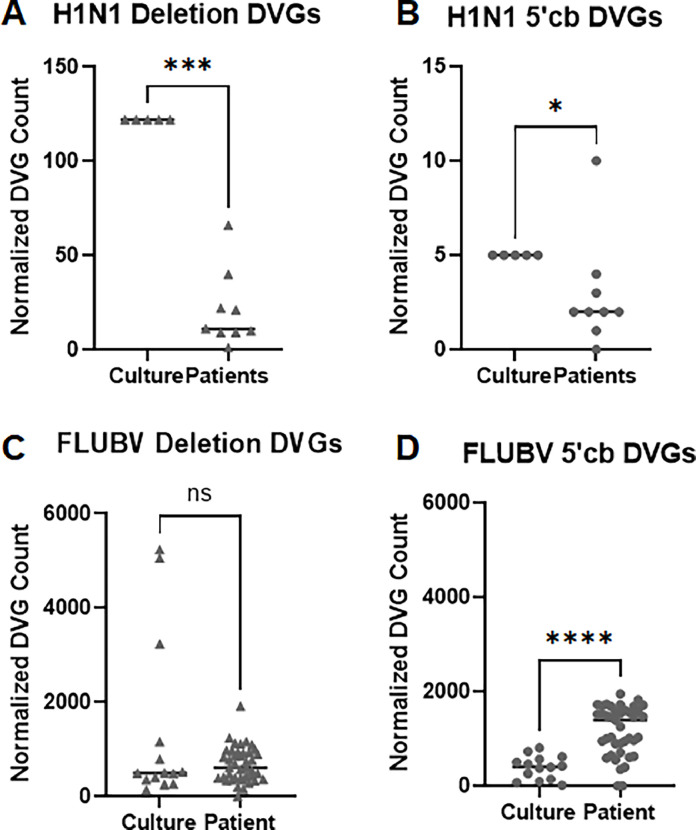
Deletion and 5′ Copyback DVGs are present in human clinical samples. DVG counts, unique junctions normalized to 1000,000 reads, are shown for FLUAV H1N1 (panels A-B from Vasilijevic et al. NGS data) and FLUBV (panels C-D from Plant et al., and Lumby et al. NGS data). Comparisons are made for DVG abundance between the cell culture and patient isolate data. Deletion DVGs are shown as triangles and 5′ cb DVGs as circles. The Mann Whitney test P values are: (A) 0.001; (B) 0.0170; (C) not significant; and (D) <0.0001.

It was not clear if the difference in DVG prevalence between the FLUAV H1N1 and FLUBV dataset is due to differences in virus type or differences in NGS data. For example, differences in the number of SRAs (Table 1) and the timing of sample collection (Lumby et al., 2020; Plant et al., 2020; Vasilijevic et al., 2017) might affect our analyses. To overcome these limitations, we analyzed additional datasets (Fig. 2). For the cell cultured virus samples we observed that the 5′ cb DVGs were less prevalent in three different FLUAV H1N1 datasets and the FLUBV dataset (Fig. 2A). For the clinical viral samples most of the datasets had significantly more 5′ cb DVGs than deletion DVGs (Fig. 2B). The notable exception is the Vasilijevic dataset for which there is significantly less 5′ cb DVGs. Subsequent analysis of the methods used to generate NGS datasets for these samples revealed that the virus was propagated in cell culture prior to RNA extraction (Vasilijevic et al., 2017). The remaining NGS datasets were generated from RNA extracted from the clinical samples without additional viral propagation (Lumby et al., 2020; Sobel Leonard et al., 2016; Xiao et al., 2019). This suggests that the abundance of 5′ cb DVGs in clinical samples can be attributed to growth *in vivo* and is not specific to the type of influenza. Interestingly, although 5′ cb DVGs were detected in dataset from zoonotic H7N9 infections, they were present at a similar level to the deletion DVGs.

**Table 1:** Sources for Next Generation Sequencing data used in study.

Influenza Type	Sample Source	BioProject ID	Reference	Number of SRAs
FLUAV H1N1	Human Patients/Culture	PRJNA327478	Vasilijevic et al. (2017)	9
FLUAV H1N1	Cell Culture	PRJNA327478	Vasilijevic et al. (2017)	5
FLUBV	Human Patient	PRJNA601176	Lumby et al. (2020)	41
FLUBV	Cell Culture	PRJNA655757	Plant et al. (2020)	14
FLUAV H1N1	Cell Culture	PRJNA725907	Alnaji et al. (2021)	3
FLUAV H1N1	Cell Culture	PRJNA601451	Barnard et al. (2021)	6
FLUAV H1N1	Human Challenge	PRJNA528931	Xiao et al. (2019)	6
FLUAV H3N2	Human Challenge	PRJNA577644	Sobel Leonard et al. (2016)	24
FLUAV H7N9	Human Patients	PRJNA202283	Unpublished	18
FLUAV H5N1	Mouse Challenge	PRJNA290088	Park et al. (2015)	9
FLUAV H5N8	Mouse Challenge	PRJNA290088	Park et al. (2015)	8
FLUAV H5N8	Ferret Challenge	PRJNA1013347	current study	4
FLUAV H5N8	Cell Culture	PRJNA1013347	current study	6
FLUAV H3N2	ANP32B-/- Mouse Challenge	PRJEB35060	Beck et al. (2020)	23
FLUAV H5N1	ANP32B-/- Mouse Challenge	PRJEB35060	Beck et al. (2020)	19

**Fig. 2:**
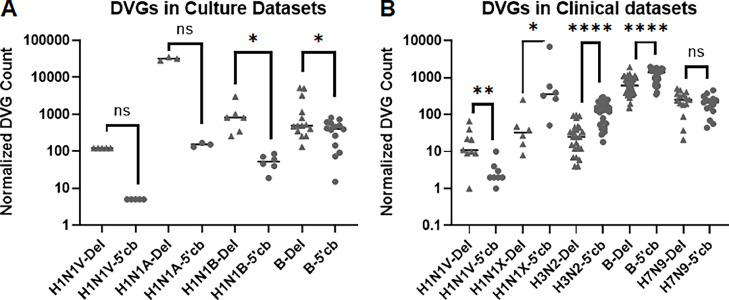
5′ Copyback DVGs are more prevalent than deletion DVGs in human clinical samples. The relative abundance of deletion and 5′ cb DVGs detected in NGS data are shown for: (A) cell cultured FLUAV H1N1 and FLUBV viruses; and (B) FLUAV H1N1, FLUAV H3N2, FLUBV and FLUAV H7N9 clinical isolates. FLUAV H1N1 samples are labeled to match source data: H1N1V from Vasilijevic et al.; H1N1A from Alnaji et al.; H1N1B from Barnard et al.; and H1N1X from Xaio et al. The FLUAV H3N2, FLUAV H7N9 and FLUBV data are from samples described in Table 1. Deletion DVGs are shown as triangles and 5′ cb DVGs as circles. The P values from Wilcoxon matched-pairs signed rank tests are: ns, not significant; *, <0.05; **, <0.01; and ****, <0.0001.

To assess whether 5′ cb DVGs were present in laboratory infected animals we analyzed the DVG composition in NGS data from FLUAV H5N1 and FLUAV H5N8 infected mice (Park et al., 2015). 5′ cb DVGs were present but, like the zoonotic H7N9 infections of humans (Fig. 2), the deletion DVG portion of the DVG population was similar the portion of 5′ cb DVGs in the mouse samples (Fig. 3). This indicates that FLUAV infected mice also produce 5′ cb DVGs but, at least in these experiments, that subpopulation did not exceed that of the deletion DVGs. The relative abundance of the different types of DVGs was similar in the H5N1 and H5N8 infected mice (Fig. 3) but differed from the pattern observed from endemic human FLUAV H1N1 and H3N2 viruses (Fig. 2). This suggests that both host species and virus species contribute to the relative abundance of the different types of DVGs. These differences warrant further investigation.

**Fig. 3:**
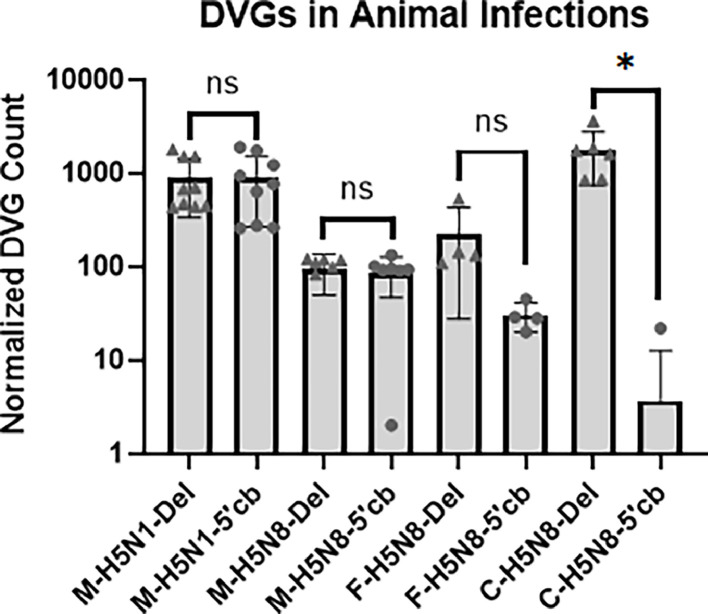
5′ Copyback DVGs are present in FLUAV infected mice and ferrets. The relative abundance of the deletion and 5′ cb DVGs detected in NGS data are shown. Samples are labeled to match source data: M-H1N1, mouse data from Park et al.; M-H5N8, mouse data from Park et al.; F-H5N8, ferret data from this work; and C—H5N8, cell culture data from this work. Deletion DVGs are shown as triangles and 5′ cb DVGs as circles. The P values from Wilcoxon matched-pairs signed rank tests are: ns, not significant; and *, <0.05.

The results described to this point compare different strains of influenza viruses. We next tested if the same input virus strain gave rise to different DVG populations when grown *in vivo* or *in vitro*. We obtained an H5N8 virus isolated in cell cultured from St. Jude Children's Research Hospital. The limited sample was diluted 1:1000 and propagated in MDCK cells and embryonated eggs. RNA was extracted from the MDCK cell culture supernatant and subjected to NGS sequencing. The titer of the egg propagated virus was determined by plaque assay and two ferrets were infected with 6.5 × 10^7^ PFU. The ferrets became ill and were euthanized on day 6 post-infection. Virus was detected in the brain and RNA was extracted from samples and subjected to NGS sequencing. The DVG profiles of the samples were compared (Fig. 3). The 5′ cb DVGs were abundant in the *in vivo* samples but not in the *in vitro* samples. This demonstrates that different types of DVGs are preferentially generated from the same virus when propagated *in vivo* or *in vitro*. The data thus far indicates that the majority of 5′ cb DVGs are generated during infection and suggests host dependency factors in the *in vivo* infections may play a role in the production of influenza DVGs.

Finally, we analyzed FLUAV DVG content from mice with defective immune systems. ANP32B has been shown to modulate inflammation in mice (Chemnitz et al., 2019). ANP32 proteins associate with influenza RdRPs and are cofactors for influenza replication (Nilsson-Payant et al., 2022; Swann et al., 2023; Zhu et al., 2023). An ANP32 deficiency in mice protects them from FLUAV disease and NGS data from samples acquired following FLUAV infection were analyzed (Beck et al., 2020). The number of normalized DVG reads was lower in ANP32B deficient mice (Fig. 4A and B). The deletion DVGs and 5′ cb DVGs appear to be similarly affected by the loss of ANP32B. This suggests that 5′ cb DVG production is diminished because viral replication is hindered rather than the ANP32 protein playing a specific role. Like the zoonotic infections described above, the abundance of 5′ cb DVGs is similar to the abundance of deletion DVGs for FLUAV H5N1 and H3N2 infections in mice.

**Fig. 4:**
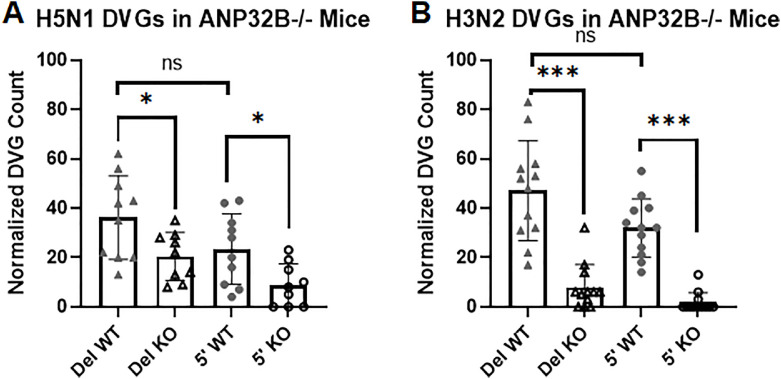
DVG abundance in ANP32B knockout mice. The relative abundance of deletion and 5′ cb DVGs is shown for (A) H5N1 infected mice and (B) H3N2 infected mice. Columns are labeled: Del for deletion DVGs; 5′ for 5′ cb DVGs; WT for wild-type mice; and KO for ANP32B-/- knockout mice. Deletion DVGs are shown as triangles and 5′ cb DVGs as circles; filled symbols are used for wild-type mouse data and open symbols for knockout mouse data. The Mann Whitney test P values between mouse groups are: ns, not significant. The P values from Wilcoxon matched-pairs signed rank tests are: *, <0.05 ; and ***, 0.0005.

Our understanding DVG production hinges on the moment the template RNA dissociates from the active site in the RdRP and when the same template, or a new template, is positioned within the RdRP to allow replication to resume. The influenza RdRPs produce three unique sets of RNA molecules during the virus lifecycle: mRNA, cRNA and vRNA. The replication of influenza cRNA or vRNA requires an additional RdRP to activate the RdRP bound to the template (Phan et al., 2021; York et al., 2013). Intriguingly, ANP32 proteins provide a link between two influenza RdRPs (Carrique et al., 2020). The arrangement of the paired RdRPs is such that newly synthesized RNA exiting one RdRP could be tracked into the template RNA entry tunnel in the second RdRP (Zhu et al., 2023). There are interesting possibilities that arise from such an arrangement. If the template RNA has dissociated from one RdRP then a nascent RNA from an adjacent polymerase could enter the stalled RdRP as a template RNA. If the adjacent RdRP provides an identical template RNA then the stalled RdRP could resume replication. If the template is positioned correctly then no aberrant product would be detected. A badly aligned template could result in insertions or deletions. If a template with the opposing sense of the original template enters the stalled RdRP then copy-back molecules could be generated. Interestingly, many of the insertions identified by DVG-profiler in our analyses were comprised of two genomic segments indicating that new templates readily replace the dissociated templates. We did not observe that 5′ cb DVGs occurred preferentially in one segment over another in any of the datasets. Further analysis is required to determine if the DVG break/join points are located in specific sequences or genomic regions.

No descriptions of 5′ cb DVGs for any influenza virus has been published prior to this work suggesting difficulties in identifying this class of DVG in influenza studies. This could be due to the nature of the virus, the DVGs themselves, or the lack tools available for detecting 5′ cb DVGs (Bosma et al., 2019). Several recent studies have highlighted the existence of diverse populations of DVGs rather than single dominant species (Boussier et al., 2020; Felt et al., 2021; Johnson et al., 2021). In contrast to other negative strand viruses with documented 5′ cb DVGs (Sendai, measles, RSV and Ebola for example), influenza genomes are both smaller and segmented. To overcome these challenges, we used datasets comprised of multiple experimental samples. However, there are some limitations in our study. Differences in NGS approaches (virus enrichment, library preparation, read depth, and sequencing chemistry) may have introduced biases. Primers used to amplify genomes could also bias the frequency of some DVGS (more DVGs with larger deletions, or more copy-back DVGs with larger loop regions for example). While technical replication of the clinical results is not feasible, the consistent prevalence of 5′ cb DVGs in clinical samples from multiple independent experiments strongly suggests 5′ cb DVGs play a role during influenza infection. We don't provide an analysis of the waxing, waning, or de novo synthesis of DVGs in the clinical FLUBV samples taken from 41 different timepoints from one patient, but do observe that, like the samples from multiple individuals infected with FLUAV H1N1 and FLUAV H3N2 (from Xiao et al., and Sobel Leonard et al. respectively), the 5′ cb population is more prevalent than the deletion DVG population.

Why 5′ cb DVGs are prevalent in the *in vivo* infections for several negative strand RNA viruses remains a mystery. There are differences in the accumulation of influenza cRNA and vRNA during infection and different approaches are used to generate the 5′ triphosphate ends: terminal initiation from the vRNA promoter, and the internal initiation and realignment from the cRNA promoter (Deng et al., 2006; Phan et al., 2021). This may contribute to the differences in the abundance or stability of 5′ cb and 3′ cb DVGs but doesn't explain the different proportion of various DVGs present during *in vitro* culture. We observe similar trends in the relative abundance of different types of DVGs from human clinical samples infected with different types of influenza virus. This pattern differs slightly from the trend observed in mice suggesting a combination of host and viral species affect the relative abundance of the different types of DVGs. Particles containing DVGs are being developed as vaccines and antivirals (Wu et al., 2022). Our results demonstrate that there are differences in the relative abundance of different types of DVGs between *in vivo* and *in vitro* experiments that must be taken into consideration during the development of treatments based on DVGs.

## Funding

This research did not receive any specific grant from funding agencies in the public, commercial, or not-for-profit sectors.

## CRediT authorship contribution statement

**Xing Li:** Investigation. **Zhiping Ye:** Supervision. **Ewan P. Plant:** Conceptualization, Data curation, Formal analysis, Writing – original draft.

## Declaration of Competing Interest

The authors declare that they have no known competing financial interests or personal relationships that could have appeared to influence the work reported in this paper.

## Data Availability

The source data is listed in Table 1.
